# Effect of transcranial direct current stimulation on postoperative delirium in elderly patients undergoing hip fracture surgery: study protocol for a randomized controlled trial

**DOI:** 10.3389/fmed.2025.1558376

**Published:** 2025-05-15

**Authors:** Li-Chao Xue, Hai-Jie Ji, Sha-Sha Fan, Qing Niu, Jun-Yan Zhang, Ai-Li Fang, Ping-Zhi Wang, Shou-Yuan Tian, Hua Zheng

**Affiliations:** ^1^Department of Anesthesiology, Third Hospital of Shanxi Medical University, Shanxi Bethune Hospital, Shanxi Academy of Medical Sciences, Tongji Shanxi Hospital, Taiyuan, China; ^2^Department of Central Laboratory, Shanxi Province Academy of Traditional Chinese Medicine & Shanxi Traditional Chinese Medical Hospital, Taiyuan, China; ^3^Department of Rehabilitation, Shanxi Bethune Hospital, Shanxi Academy of Medical Sciences, Tongji Shanxi Hospital, Third Hospital of Shanxi Medical University, Taiyuan, China; ^4^Department of Clinical Epidemiology and Evidence-based Medicine, Shanxi Bethune Hospital, Shanxi Academy of Medical Sciences, Tongji Shanxi Hospital, Taiyuan, China; ^5^Department of Anesthesiology, Shanxi Provincial People's Hospital, Shanxi Medical University, Taiyuan, China; ^6^Department of Anesthesiology, Hubei Key Laboratory of Geriatric Anesthesia and Perioperative Brain Health, and Wuhan Clinical Research Center for Geriatric Anesthesia, Tongji Hospital, Tongji Medical College, Huazhong University of Science and Technology, Wuhan, China

**Keywords:** hip fracture, post-operative delirium, transcranial direct current stimulation, quality of recovery, clinical protocols

## Abstract

**Objective:**

To evaluate the efficacy of transcranial direct current stimulation (tDCS) in reducing the incidence of postoperative delirium (POD) in elderly patients undergoing hip fracture surgery.

**Methods and analysis:**

This single-center, double-blind, randomized controlled trial will enroll 160 participants aged 65 years and older, scheduled for elective hip surgery under spinal anesthesia. Participants will be randomly assigned to either the active-tDCS group or the sham-tDCS group. The active-tDCS group will receive two sessions: one pre-surgery and one post-surgery, with electrodes positioned over the left dorsolateral prefrontal cortex and the right supraorbital area. Each session includes 15-second ramp-up phase at the start, 20 min simulation with 2 mA current and 15-second ramp-down phase at the end. The sham-tDCS group will receive two sham procedures with no actual current delivered. Functional brain activity will be monitored before and after each session or sham procedure to assess changes in cortical activation and connectivity using functional near-infrared spectroscopy (fNIRS). The primary outcome measure will be the incidence of POD, assessed using the 3-Min Diagnostic Interview for Confusion Assessment Method (3D-CAM). Secondary outcomes include the severity of delirium, postoperative pain, anxiety, depression, cognitive function, and sleep quality.

**Trial registration:**

The trial was registered at ClinicalTrials.gov (NCT06678529) on Oct 22, 2024.

## Introduction

Post-operative delirium (POD) is a common complication in elderly patients (1). The incidence of POD varies by surgical type and invasiveness. More invasive, extensive procedures typically lead to higher POD rates than less invasive, shorter ones (2). POD can affect the prognosis of patients and even increase the mortality rate. However, as of now, the available effective treatments for postoperative delirium remain limited. Hence, it is crucial to identify efficacious interventions that can prevent this condition.

Transcranial direct current stimulation (tDCS) is a non-invasive neuromodulation technique that can alter cortical excitability and the spontaneous electrical activity of neurons (3). This method has been shown to enhance the functional connectivity of key regions within the left frontoparietal sensorimotor network, as well as neural circuits involved in consciousness regulation (4). Evidence supports the use of tDCS in improving various cognitive functions, including memory, attention, and perception (5). A study by Tao et al. demonstrated that a single session of left dorsolateral prefrontal tDCS significantly reduced the incidence of postoperative delirium in elderly patients undergoing lower limb joint replacement under general anesthesia, with rates decreasing from 19.7% to 4.9% (6). Functional near-infrared spectroscopy (fNIRS) is an emerging brain imaging modality that offers high spatial and temporal resolution, along with resistance to motion artifacts and electromagnetic interference (7). It is a safe, non-invasive, and user-friendly technology that enables real-time monitoring, making it particularly promising for applications in neurocognitive assessment, clinical diagnostics, and rehabilitation (8). fNIRS works by quantifying changes in blood oxygenation within brain tissue, using the absorption and scattering of near-infrared light, with data processed via the Beer-Lambert Law (9). This technique indirectly reflects neural activity, enabling real-time evaluations of brain function, and may serve as a biomarker for the early detection of delirium, offering a novel tool for monitoring postoperative cognitive status.

In elderly patients, particularly those with cardiopulmonary comorbidities, spinal anesthesia is increasingly preferred over general anesthesia for hip fracture surgery, although there is no significant difference in the effect of spinal anesthesia and general anesthesia on postoperative delirium (10, 11). The aim of this study is to conduct a randomized, controlled, double-blind trial to (1) evaluate the potential of tDCS in reducing the incidence of postoperative delirium in elderly patients undergoing hip fracture surgery under spinal anesthesia, and (2) use fNIRS to investigate the mechanisms through which tDCS modulates brain network connectivity, brain region activation, and the reduction of POD. This investigation seeks to provide clinical evidence supporting the use of tDCS as an intervention to mitigate postoperative delirium in this vulnerable patient population.

## Materials and methods

### Study design

**Figure 1:**
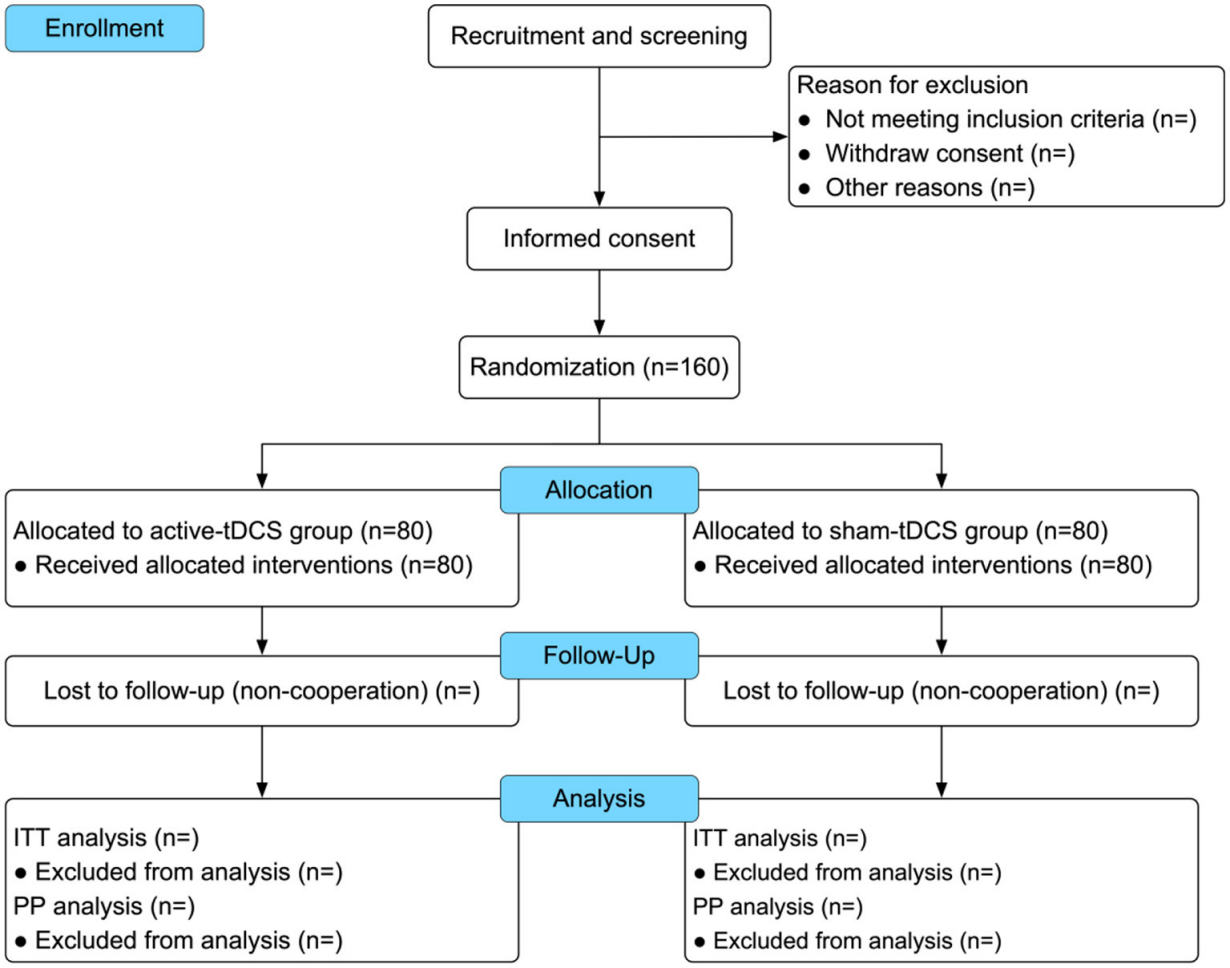
Trial flow chart. tDCS, transcranial direct current stimulation; ITT, intention-to-treat; PP, per-protocol.

This study is a prospective, single-center, double-blind, randomized controlled trial, conducted in accordance with the Standard Protocol Items: Recommendations for Interventional Trials (SPIRIT) guidelines (12). The trial staff will review orthopedic inpatient registration lists and elective surgery application forms to identify adults aged 65 years and older scheduled for surgical repair of clinically or radiologically diagnosed femoral neck, intertrochanteric, or sub-trochanteric hip fractures. Potential participants will be screened for eligibility based on inclusion and exclusion criteria, which will be assessed through medical record reviews and face-to-face interviews. The study design is depicted in Figure 1, and the trial schedule is outlined in Table 1.

**Table 1:** Time schedule of the trial process.

**Item**	**T0**	**Pre-surgery**			**Post-surgery**						

		**f1**	**1st tDCS**	**f2**	**f3**	**2nd tDCS**	**f4**	**Day 1**	**Day 2**	**Day 3**	**Day 5**
Enrolment											
Informed consent	×										
Eligibility screen	×										
Demographics	×										
Diagnosis	×										
Comorbidities	×										
Frailty scale	×										
ASA grade	×										
MMSE	×										×
GAD-7	×							×		×	
PHQ-9	×							×		×	
Laboratory tests	×										
Randomization	×										
Interventions		×	×	×	×	×	×				
Assessments											
3D-CAM^*^	×							×	×	×	
NRS	×							×	×	×	×
Analgesia								×	×	×	
PSQI	×							×	×	×	
Complications											×

The four functional near-infrared spectroscopy (fNIRS) assessments will be conducted at specific time points before and after transcranial direct current stimulation (tDCS) as follows: (1) pre-surgery, pre-tDCS (f1): the first fNIRS measurement will be obtained prior to surgery, before the first application of tDCS; (2) pre-surgery, post first tDCS (f2): the second fNIRS measurement will be performed before surgery, following the first application of tDCS; (3) post-surgery, before second tDCS (f3): the third fNIRS measurement will be conducted after surgery, before the second application of tDCS; (4) post-surgery, post second tDCS (f4): the fourth fNIRS measurement will be taken after surgery, following the second application of tDCS. ASA, American Society of Anesthesiologists; MMSE, Mini-Mental State Examination; GAD-7, Generalized Anxiety Disorder-7; 3D-CAM, 3-min diagnostic interview for Confusion Assessment Method; PHQ-9, Patient Health Questionnaire-9; ^*^indicates twice a day, once in the morning and once in the afternoon; NRS, Numeric Rating Scale; PSQI, Pittsburgh Sleep Quality Index.

### Ethics approval and consent

The study will be conducted in full compliance with relevant regulations, including the Declaration of Helsinki as set forth by the World Medical Association. Prior to initiation, the research protocol will be reviewed and approved by the Institutional Review Board. Before enrollment, the investigators will thoroughly explain the study's objectives, procedures, and potential risks to each participant or their legal representative. Written informed consent will be obtained from all participants, ensuring they understand their right to withdraw from the study at any time without consequence. Informed consent documents will be retained as part of the clinical research records. Throughout the duration of the study, participant privacy and data confidentiality will be strictly maintained. This study has been approved by the Institutional Review Board of Shanxi Bethune Hospital (approval number YXLL2024191) and is registered on ClinicalTrials.gov (registration number NCT06678529).

### Inclusion criteria

Participants will eligible to participate in this study after meeting all of the following criteria: (1) aged ≥ 65 years; (2) scheduled for elective hip fracture, including femoral head fractures, femoral neck fractures, intertrochanteric or sub-trochanteric fractures, femoral head replacement, total hip arthroplasty, or open or closed reduction; (3) platelet count >80 × 10^9^/L; (4) ASA classification ≤ Grade III; (5) Mini-Mental State Examination (MMSE) scores ≥ 18 points (13); (6) willingness to participate and sign the informed consent form.

### Exclusion criteria

Participants will be excluded from this study if they meet any of the following criteria: (1) severe mental disorders (e.g., depression or schizophrenia requiring medication treatment); (2) cranial or scalp injuries; (3) history of symptomatic cerebrovascular disease, including stroke, transient ischemic attack; (4) compound injuries, multiple fractures, periprosthetic fractures, and hip joint revisions; (5) drug or alcohol abuse; (6) severe visual or hearing impairments; (7) history of epilepsy or intracranial metal implants; (8) severe cardiovascular disease history and liver dysfunction, or kidney dysfunction; (9) coagulation abnormalities; (10) severe chronic obstructive pulmonary disease; and (11) participation in other clinical studies within the past 3 months.

### Termination criteria

The study will be terminated if a participant experiences conditions that make it inappropriate to continue their involvement in the study, including: (1) worsening of the participant's medical condition; (2) severe adverse events; and (3) poor compliance with study protocols. Additionally, the study may be terminated under the following circumstances: (1) discovery of serious safety issues by investigators; (2) substantial flaws or errors in the study protocol that affect the study's integrity or participant safety; (3) the sponsor decides to terminate the study due to financial constraints or management issues; and (4) administrative authorities revoke approval for the trial. Termination of the study may be either temporary or permanent. In the event of termination, all trial records must be preserved and retained for future reference.

### Randomization and blinding

An independent research coordinator will oversee randomization and group allocation. Randomization will be performed using Stata statistical software, and allocation concealment will be ensured through the sealed envelope method. Sequentially numbered, opaque black envelopes containing random allocation numbers will be prepared and stored at the research site. Enrolled participants will receive the envelope with the lowest available number, corresponding to their group assignment. On the day of surgery, after the participant has been admitted to the Post - Anesthesia Care Unit (PACU), the clinical research coordinator will open the assigned envelope, note the allocation, and assign the participant to the appropriate study group. The participant's identification number will be recorded. The physician administering tDCS will be informed of the participant's group allocation. This process will be repeated for each subsequent participant. Once an envelope has been opened, it will not be reused. If a participant withdraws from the study after the envelope has been opened, their data will still be included in the analysis, and the reason for withdrawal will be documented. All participants will remain blinded to their group assignment. Surgeons, anesthesiologists, and ward and operating room nurses will also be blinded to the group allocations and intervention protocols. Additionally, personnel involved in outcome assessment, follow-up, data collection, and data processing will remain blinded to both the intervention protocols and group assignments.

### Emergency unblinding

In the event of a severe adverse event occurring in a participant, research personnel must immediately inform the principal investigator to assess and confirm the situation. If emergency unblinding is deemed necessary, the intervention research personnel must promptly notify the research coordinator. The research coordinator will then access the participant's group allocation and specific intervention details to facilitate the emergency unblinding process. Upon unblinding, the research coordinator will communicate the participant's group assignment to the intervention research personnel, who will proceed with managing the participant's treatment according to their allocated group. The research coordinator must also complete an emergency unblinding record promptly, documenting the reason for the unblinding and any relevant details associated with the event. This record will be maintained as part of the study's documentation.

### Interventions

**Figure 2:**
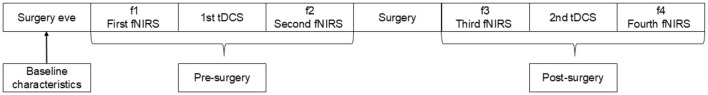
Timeline of the experimental design. tDCS, transcranial direct current stimulation; fNIRS, functional near-infrared spectroscopy.

The standard intervention consists of two sessions of tDCS and four sessions of fNIRS. The intervention timeline is depicted in Figure 2. The tDCS sessions will be administered on the day of surgery: the first session will be conducted preoperatively and the second session will take place postoperatively in the PACU. The fNIRS assessments will be performed as part of the monitoring protocol at four designated time points throughout the study.

The tDCS procedure is administered using a transcranial direct current stimulator, with two electrodes placed in physiological saline soaked sponges. The electrodes are secured with a stretchable cap, positioning the anode over the left dorsolateral prefrontal cortex (DLPFC) and the cathode over the right supraorbital area. The active-tDCS group will receive a 15-second ramp up phase at the start, 2 mA of tDCS for 20 min and a 15-second ramp down phase at the end. The sham-tDCS group will only receive the 15-second ramp up phase at the beginning and the 15-sencond ramp down phase at the end of each session, without the continuous 2 mA current for 20 min (6). Research personnel administering the intervention will closely monitor participants for any discomfort. If a participant experiences any intolerable discomfort, the stimulation will be immediately terminated.

Four fNIRS procedures will be conducted in relation to the tDCS interventions: (1) f_1_ (pre first tDCS) will be conducted before the first tDCS session; (2) f_2_ (post first tDCS, pre-surgery) will be conducted after the first tDCS session and before surgery; (3) f_3_ (post-surgery, before second tDCS) will be conducted after surgery and before the second tDCS session; (4) f_4_ (post second tDCS) will be conducted after the second tDCS session. The fNIRS device used is the NirSmartII-6000 A, a 41-channel system that includes 19 light emitting source probes and 16 detector probes. The system comprises near infrared light sources and avalanche photodiodes as detectors, with a sampling frequency of 11 Hz. This system continuously measures and records changes in the concentrations of oxygenated hemoglobin (HbO) and deoxygenated hemoglobin (HbR) in the brain during task performance. Relative concentration changes in HbO, HbR, and total hemoglobin (HbT) are calculated based on the modified Beer Lambert Law.

Data collection during resting state and Verbal Fluency Task (VFT): during the resting state, the concentrations of HbO and HbR across the entire cerebral cortex are recorded. In the resting state participants are required to remain quiet, awake, and relaxed, avoiding any specific mental activities or physical movements, and lie in a supine position for 5 min. During the VFT, the concentrations of HbO and HbR in the prefrontal and temporal cerebral cortex are recorded. The VFT consists of three phases: (1) pre-task phase: participants count from 1 to 5 for 30 seconds; (2) task phase: participants are presented with three Chinese characters and are instructed to form as many words as possible using each character within 20 seconds per character; and (3) post task phase: participants again count from 1 to 5 for 70 seconds. The VFT is administered via computer, and participants are instructed to remain as quiet and undisturbed as possible throughout the task.

Throughout the intervention, standard anesthesia monitoring is maintained, including non-invasive blood pressure measurement, electrocardiogram, and pulse oximetry to ensure participant safety. The administering physicians and research personnel are trained to recognize and respond promptly to any adverse events. All equipment is calibrated and maintained according to manufacturer specifications to ensure accurate and reliable data collection. Data from participants who are unable to receive both tDCS sessions for any reason will still be included in the intention to treat analysis to preserve the randomization benefits and provide an unbiased estimate of the intervention effect.

### Anesthesia procedures

Upon entering the operating room, an intravenous access is established, and routine oxygen supplementation is provided via nasal cannula. Standard anesthetic monitoring procedures are implemented, including non-invasive blood pressure monitoring, electrocardiogram monitoring, and pulse oximetry. Prior to positioning, 5 μg/kg of alfentanil are administered intravenously to alleviate pain associated with positional changes. The patient is positioned with the affected side facing upwards, and 3 ml of 2% lidocaine hydrochloride is used for local infiltration at the puncture site. Puncture is performed at the L2/3 or L3/4 intervertebral space, with clear cerebrospinal fluid observed upon puncture. Following this, 20 mg of ropivacaine hydrochloride is injected, with aspiration before and after injection to confirm the correct placement. Five min after the injection, light touch sensation is tested to assess the anesthetic level, which should reach the T10 dermatome. After confirming proper anesthesia, sterile draping is applied, and the surgical procedure is initiated.

If the surgical duration is prolonged and the patient exhibits signs of nervousness or discomfort, a small dose of etomidate lipid emulsion injection may be administered for short-term maintenance to ensure patient comfort during the procedure. Alternatively, other sedative medications may be used at the discretion of the attending anesthesiologist. Vasoactive agents are administered to maintain blood pressure and heart rate within ±20% of baseline values. Additionally, appropriate warming measures are implemented to prevent hypothermia. Postoperatively, patient-controlled analgesia (PCA) is administered using sufentanil 1.5 μg/kg and palonosetron 0.25 mg diluted in 100 ml of saline. The PCA pump is configured with a continuous infusion rate of 2 ml/h, a bolus dose of 0.5 ml, and a lockout interval of 15 min (6).

### Data collection

#### Baseline characteristics

After enrollment, the following baseline characteristics will be recorded for each subject: age, gender, height, body mass index (BMI), years of education, and preoperative comorbidities including hypertension, coronary heart disease (with a documented history), arrhythmia (defined as sinus bradycardia, sinus arrest, atrial fibrillation, and other arrhythmias requiring medication intervention), history of stroke (including ischemic stroke and hemorrhagic stroke), obesity (defined as BMI ≥ 30 kg/m^2^), Charlson comorbidity index, ASA classification, MMSE score, frailty screening scale, anxiety and depression scores, anesthesia and surgical history. Preoperative laboratory tests will include serum albumin, hemoglobin, C-reactive protein, neutrophils/lymphocytes, and type of surgery (hip replacement, open reduction and internal fixation, or closed reduction).

#### Intraoperative data

Duration of the surgery (defined as from skin incision to skin closure), duration of anesthesia (defined as from the start of monitoring after entering the operating room to the cessation of monitoring), whether nerve block is administered, total amount of anesthetic drugs used, including sedatives, dexamethasone, and vasopressor usage. volume of crystalloid and colloid fluids used during surgery, estimated blood loss, and intraoperative blood transfusion.

#### PACU

In the PACU, the intervention will be provided, and vital signs will be observed and recorded. Possible adverse reactions, such as skin tingling, mild fatigue, itching, burning sensation, skin erythema, and mild headache, will also be documented (14, 15). The fNIRS data will be processed and analyzed using the Nir Spark software package v1.7.3 (16). Initially, the raw data will be converted into optical density signals, and motion artifacts will be corrected using a three-sample interpolation method. Subsequently, the obtained signals will undergo bandpass filtering (0.01–0.2 Hz) to eliminate physiological noise from pulsation, respiration, and other sources of physiological fluctuations. For the brain activation analysis of during the VFT paradigm, a General Linear Model (GLM) will be employed to analyze the time series data of HbO and HbR. A *t* test will be conducted on the baseline and task state signals for each channel of each participant. A typical Hemodynamic Response Function (HRF) with temporal and dispersion derivatives will be selected as the fundamental function for the GLM. By calculating the degree of match between the experimental HRF values and the design values, the GLM will generate an activation coefficient (β value) representing the intensity of task induced cortical activation in the participants' brains. For HbO signals, a positive β value indicates an increase in task related cortical activation, while a negative β value indicates a decrease. For brain network analysis of resting state paradigm, Pearson correlation analysis will be performed on the time series of each pair of channels. For each participant, A 41 × 41 correlation matrix will be generated for each participant to explore the correlation between cortical activity and behavioral performance.

### Follow-up

Delirium will be assessed during the first three post-operative days, along with pain at rest and during movement and the dosage of analgesics. On the fifth post-operative day, assessments will include the MMSE and the occurrence of any complications. The primary endpoint of the study is the completion of cognitive evaluation on postoperative day five. Delirium will be assessed by trained specialists, who will undergo a rigorous training and receive comprehensive guidance from expert psychiatric consultants prior to the commencement of the trial. Delirium will be evaluated twice daily during the first three post-operative days. The assessment time points will be scheduled from 9:00 AM to 11:00 AM and 3:00 PM to 5:00 PM, ensuring a minimum interval of 6 h between assessments. POD will be assessed using a 3-min diagnostic interview (3D-CAM) conducted by researchers who were blinded to the study group allocation. The Confusion Assessment Method (CAM) assessment is based on the four key features of delirium: acute onset and fluctuating course, disorganized thinking, inattention, and altered levels of consciousness. Investigators will also consult the medical record or contact a family member, friend, or health care provider who knows the patient well to gather additional information regarding the patient's symptoms. If POD is identified, daily assessments will continue until symptoms resolve or the patient is discharged. Delirium subtypes will be assessed using the Richmond Agitation Sedation Scale (RASS) (17). Hyperactive delirium is characterized by symptoms ranging from mild restlessness to persistent motor activity and irritability. Conversely, hypoactive delirium is characterized by one or more of the following features: reduced or absent movement, minimal verbal communication despite stimulation, and a lack of responsiveness. Mixed delirium is characterized by rapid fluctuations between the hyperactive and hypoactive states. The severity of delirium will be assessed using the Delirium Rating Scale Revised-98 (DRS-R-98) (18).

Pain at rest and during movement will be assessed using the Numeric Rating Scale (NRS) (19), an 11-point scale where 0 denotes no pain and 10 represents the worst possible pain. Anxiety will be evaluated using the Generalized Anxiety Disorder-7 (GAD-7) (20). The total score, ranging from 0 to 21, is calculated by summing up the individual item scores. A total score of 10 or higher is considered a positive result. Conversely, a score below 10 is regarded as a negative result. Depression severity will be assessed using the Patient Health Questionnaire-9 (PHQ-9) (21). Scores ranging from 0 to 4 indicate of normalcy, while scores between 5 and 9, and 10 to 14, suggest mild and moderate depression, respectively. Scores between 15 and 19, and 20 to 27, indicate moderately severe and severe depression, respectively. The Pittsburgh Sleep Quality Index (PSQI) is used to evaluate sleep quality, with scores ranging from 0 to 21. Higher scores suggest poorer sleep quality (22). The MMSE is a well-established, standardized, simple and convenient tool for cognitive screening and assessment (23). The examination encompasses six key domains: orientation, attention, calculation, memory, language, and visuospatial ability.

### Data quality and management

After screening and enrollment, participants' names will be replaced with unique four-letter codes to ensure privacy protection. Paper based data will be recorded in Case Report Forms (CRFs), which will include demographic information, medical data, intraoperative management details, post-operative follow-up data, and adverse event documentation. All collected data will be securely stored in Department of Anesthesiology, Shanxi Bethune Hospital.

### Clinical outcomes and assessments

The primary outcome of this study is the occurrence rate of delirium on post-operative days 1, 2, and 3. Secondary outcomes include the severity of POD, pain scores at rest and during movement, anxiety and depression scores, sleep quality, as well as post-operative cognitive function and complications. Another key outcome is the change of whole brain cortical networks and brain activation patterns during resting state and verbal fluency tasks.

### Sample size

We hypothesize that two sessions of tDCS will reduce the incidence of postoperative delirium compared to the sham stimulation group. A meta-analysis published in 2023 on the incidence of delirium following non-cardiac surgeries in China reported an approximate postoperative delirium rate of 20% (24). The study by Tao et al. demonstrated that a single session of tDCS postoperatively reduced the incidence of delirium in elderly patients undergoing lower limb joint replacement under general anesthesia, decreasing it from 19.7% to 4.9% (6). Therefore, we conducted a sample size calculation based on a two-sided chi-square test, using a significance level of 0.05 and a power of 0.8, which indicated that 71 participants per group are required. Considering a 10% loss to follow-up rate observed in previous studies, the final sample size was set at 80 participants per group.

### Statistical analysis

The Kolmogorov-Smirnov test will be used to assess the normality of the data. For continuous variables that follow a normal distribution, the mean and standard deviation (SD) will be reported, while non-normally distributed variables will be expressed as median and interquartile ranges (IQRs). Independent sample *t*-tests will be used for normally distributed data, and the Mann-Whitney U test will be applied for non-normally distributed data. For the binary outcomes, chi square test or fisher exact test will be implemented.

The difference between-groups on the primary outcome will be reported as relative risk (RR) with 95% confidence intervals (CI), and the *p*-value < 0.05 will be considered statistically difference. All *p*-values for secondary outcomes and other exploratory analyses will be calculated to explore their potential impact on the overall study conclusions. These *p*-values will be considered nominal and should be interpreted as descriptive.

If patients sign the informed consent but refuse the assigned intervention, they will still be included in the statistical analysis, and patients who refuse the delirium assessment, they will be considered as having delirium. The study will use both the Intention-to-Treat (ITT) and Per-Protocol (PP) populations for statistical analysis. SPSS 26.0 (IBM Corp, Armonk NY) will be used for statistical analyses, with a 2-sided and *p*-value < 0.05 considered statistically significant.

## Discussion

This randomized, double-blind, single center clinical trial investigates the effects of tDCS on POD in elderly patients undergoing hip fracture surgery under spinal anesthesia. Compared to sham stimulation, active tDCS is expected to effectively reduce the incidence and severity of POD while enhancing frontal cortical activity. Previous studies have demonstrated that tDCS can improve cognitive function in post-stroke patients, enhancing working memory, attention, and daily activity performance (25, 26). This study will provide theoretical support for the clinical application of tDCS and offer a safe and effective strategy for the prevention or early intervention of delirium.

fNIRS is used to explore the mechanisms underlying POD, which contains the following features: (1) simplicity and versatility: fNIRS is a simple, portable, and highly adaptable tool that has been widely applied in stroke rehabilitation, motor function studies, learning, and mental health research (27); (2) naturalistic monitoring: fNIRS enables researchers to monitor brain activity under simulated real-world conditions, allowing participants to perform cognitive tasks and resting state imaging in an open, patient-friendly, and clinical care environment (28); (3) safety and cost-effectiveness: fNIRS is a safe and relatively inexpensive imaging technique. Compared to other neuroimaging modalities, such as functional magnetic resonance imaging (fMRI), it offers superior temporal resolution and is well-suited for studying of dynamic brain activity (29).

In summary, this study highlights the potential of tDCS as an effective and safe intervention for reducing POD in elderly patients undergoing hip fracture surgery. By integrating fNIRS imaging, the research provides novel insights into the neural mechanisms underlying POD and contributes to the development of innovative rehabilitation strategies. Future multicenter trials with larger sample sizes are recommended to validate these findings and explore the broader applicability of this intervention.
